# The Importance of Succinylacetone: Tyrosinemia Type I Presenting with Hyperinsulinism and Multiorgan Failure Following Normal Newborn Screening

**DOI:** 10.3390/ijns6020039

**Published:** 2020-05-16

**Authors:** Jessica R. C. Priestley, Hana Alharbi, Katharine Press Callahan, Herodes Guzman, Irma Payan-Walters, Ligia Smith, Can Ficicioglu, Rebecca D. Ganetzky, Rebecca C. Ahrens-Nicklas

**Affiliations:** 1 Department of Pediatrics, Division of Human Genetics, Section of Biochemical Genetics, Children’s Hospital of Philadelphia, Philadelphia, PA 19104, USA; alharbih@email.chop.edu (H.A.); payan@email.chop.edu (I.P.-W.); smithl16@email.chop.edu (L.S.); ficicioglu@email.chop.edu (C.F.); ganetzkyr@email.chop.edu (R.D.G.); ahrensnicklasr@email.chop.edu (R.C.A.-N.); 2 Department of Pediatrics, Pediatric Residency Program, Children’s Hospital of Philadelphia, Philadelphia, PA 19104, USA; guzmanh@email.chop.edu; 3 Department of Pediatrics, Division of Neonatology, Children’s Hospital of Philadelphia, PA 19104, USA; callahankp@email.chop.edu; 4 Department of Pediatrics, University of Pennsylvania Perelman School of Medicine, Philadelphia, PA 19104, USA

**Keywords:** tyrosinemia type I, hereditary tyrosinemia, newborn screening, succinylacetone, Tyrosine

## Abstract

Tyrosinemia type I (TT1) is an inborn error of tyrosine metabolism with features including liver dysfunction, cirrhosis, and hepatocellular carcinoma; renal dysfunction that may lead to failure to thrive and bone disease; and porphyric crises. Once fatal in most infantile-onset cases, pre-symptomatic diagnosis through newborn screening (NBS) protocols, dietary management, and pharmacotherapy with nitisinone have improved outcomes. Succinylacetone provides a sensitive and specific marker for the detection of TT1 but is not universally utilized in screening protocols for the disease. Here, we report an infant transferred to our facility for evaluation and management of hyperinsulinism who subsequently developed acute-onset liver, respiratory, and renal failure around one month of life. She was found to have TT1 caused by novel pathogenic variant in fumarylacetoacetate hydrolase (c.1014 delC, p.Cys 338 Ter). Her NBS, which utilized tyrosine as a primary marker, had been reported as normal, with a tyrosine level of 151 µmol/L (reference: <280 µmol/L). Retrospective analysis of dried blood spot samples via tandem mass spectrometry showed detectable succinylacetone ranging 4.65–10.34 µmol/L. To our knowledge, this is the first patient with TT1 whose initial presenting symptom was hyperinsulinemic hypoglycemia. The case highlights the importance of maintaining a high suspicion for metabolic disease in critically ill children, despite normal NBS. We also use the case to advocate for NBS for TT1 using succinylacetone quantitation.

## 1. Introduction

### 1.1. Tyrosinemia Type I

Gentz and colleagues first described tyrosinemia in 1965 in a cohort of children with liver failure, cirrhosis, and renal dysfunction with high levels of tyrosine in their urine and plasma [1]. The defect was later found to arise from decreased fumarylacetoacetase (fumarylacetoacetate hydrolase; FAH) activity, resulting in toxic accumulation of tyrosine metabolites fumarylacetoacetate and succinylacetone (SUAC) [2]. Worldwide, the incidence of tyrosinemia type I (TT1, hereditary tyrosinemia, FAH deficiency; MIM #276700) is about 1:100,000 births [3], with increased incidence in certain populations across the globe [4], most notably in Quebec [5] and Scandinavia [6,7].

Untreated, TT1 was often fatal secondary to liver pathology, including liver failure, recurrent bleeding, or hepatocellular carcinoma (HCC), or to neurologic crises [8,9]. Among children who presented symptomatically prior to 2 months of age and managed only with dietary restriction of tyrosine and phenylalanine, 2-year survival rates were 29%, while 2-year survival rates were 74% for those who presented between 2 and 6 months of age [9] Despite this improvement in short-term survival, all children presenting prior to 6 months of age had died by 12 years of age if they had not undergone liver transplantation (LT) [9]. The majority of TT1 cases present prior to 6 months of age, with variable periods of time between symptom onset and diagnosis [9]. Among children presenting after 6 months of age, 2-year survival rates were 96%. However, even in this latter group 4/10 children did not survive to their 10th birthday [9]. LT improves survival rates by correcting liver pathologies but is not free from accompanying morbidity and mortality. Five-year survival rates for LT in TT1 have ranged from 70% (7/10) in patients studied 1981–1987, prior to the availability of pharmacotherapy [10] to 90% (113/125) in patients studied 1987–2008 [11]. In the former study, all but one patient was managed with dietary restriction prior to LT. In the latter study, there was no improvement in survival over time [11], suggesting against improved outcomes due to the introduction of pharmacotherapy or screening practices alone.

In 1992, Lindstedt and colleagues reported successful use of the 4-hydroxyphenylpyruvate dioxygenase inhibitor 2-(2-nitro-4-trifluoromethylbenzoyl)-1, 3-cyclohexanedione (NTBC; nitisinone; brand-names Orfadin or Nityr) in the treatment of 5 patients with TT1 [12]. Compared with both late (after one month of age) or no therapy, the Quebec NTBC Protocol showed that early (prior to one month of age) initiation of NTBC eliminated acute manifestations of TT1 and reduced the need for LT with only rare side-effects—most notably, corneal deposits [13]. A French study showed similarly-improved outcomes with NTBC therapy in 45 patients followed for an average of 4 years 9 months, but notably reported one infant who did not respond to NTBC [14]. The Gothenburg NTBC Trial found 6 patients (10%) failed to clinically improve on NTBC [15]. Presently, NTBC pharmacotherapy in conjunction with a protein-restricted diet are standard of care for patients diagnosed with TT1 [16,17].

It is important to note that the effects of pre-symptomatic NTBC initiation are difficult to separate from the effects of initiation at the onset of symptoms in studies with patients spanning multiple decades during which the standard of care for TT1 management has changed dramatically and for which long-term follow-up has not been possible. However, it remains widely accepted that pre-symptomatic diagnosis of TT1 plays an important role in preventing morbidity and mortality, making a robust newborn screening (NBS) protocol for the disease desirable [16,17,18]. Experience in Quebec showed that no patients detected by NBS and treated according to the study protocol developed HCC, renal disease, or neurologic crisis with follow-up periods exceeding 10 years [13,19]. In 6 patients not identified by NBS due to either birth outside of Quebec or screening failure, 4 required LT, despite the initiation of NTBC following diagnosis [13]. When 12 children in the UK were tested for TT1 at birth and initiated on NTBC at an average age of four days, none demonstrated laboratory or imaging evidence of liver pathology at 8.5 years follow-up [20]. Authors concluded that NBS for TT1 should be universal [20]. In Spain, 7/8 patients diagnosed via NBS at an average age of 12.7 days and initiated on NTBC pre-symptomatically remained liver- and renal-disease free at an average follow-up time of 6.1 years [21]. The remaining patient presented in acute liver failure, but also remained liver- and renal-disease free at the time of follow-up [21]. While there was no difference in poor hepatic outcomes between patients diagnosed via NBS and those diagnosed clinically (*p*-value of 0.06), there was a difference in cognitive outcomes, with the latter group demonstrating lower IQs or psychomotor developmental indices on average (*p*-value 0.009) [21].

### 1.2. Screening for Tyrosinemia Type I

As early as 1977, SUAC was recognized to be present in fresh urine samples from patients with TT1 [2]. Authors found no SUAC in samples from either healthy patients or those with a variety of non-TT1 illnesses, including severe liver disease [2]. As the compound is not an intermediate of any known metabolic pathway, this work provided the first clue that SUAC might prove to be a useful screening marker for TT1 [2]. Today, the presence of blood SUAC is considered pathognomonic for a TT1 diagnosis.

In 1982, measurement of SUAC in amniotic fluid permitted prenatal diagnosis of TT1 [22] and the same group simultaneously published on the utility of gas chromatography/mass spectrometry in screening dried blood spots for patients with TT1 [23]. Such methods relied on indirect SUAC quantitation following enzymatic inhibition of δ-aminolevulinic acid dehydratase. Schulze et al. suggested the assay more optimal for second-tier screening as false-positive results could be obtained in the setting of high temperatures, genetic differences in enzyme activity, or theoretically lead exposure [24] While indirect SUAC quantitation was utilized in Quebec’s NBS program as a second-tier test following tyrosine quantitation 1970–1997 and as a first-tier test 1998–2014 [23,25], it was not more widely adopted. In 2004, Allard and colleagues developed a method allowing direct SUAC quantitation from dried blood spots using tandem mass spectrometry (MS/MS) [26]. Shortly thereafter, methods allowing quantitation of SUAC combined with amino acids and acylcarnitines were developed, which helped to improve screening efficiency by reducing the need for additional laboratory preparation or equipment [27]. Quebec transitioned to direct SUAC quantitation via MS/MS as the primary marker in TT1 screening in 2014 [25]. Since then, 14/14 TT1 cases were identified by NBS with 5 false positive results, yielding a sensitivity of 100% and a positive predictive value of 73.6% [25].

Tyrosine-dependent methods have both poor sensitivity and specificity for FAH deficiency, depending on screening thresholds values. Greater than half of TT1 patients have NBS tyrosine levels that fall within the control range [28] La Marca and colleagues reported two infants with TT1 diagnoses who demonstrated normal tyrosine levels and were identified through SUAC-based testing methods [29,30]. However, given the degree of overlap in tyrosine levels between cases and controls [28], it is likely that the true incidence of cases unidentified by tyrosine-based screening is higher. The sensitivity of TT1 screening using SUAC detection was 100% across five studies [26,27,30,31,32] involving 29 cases and over 35,000 controls systematically reviewed by Stinton et al., although each study employed a slightly different SUAC cutoff [33]. Specificity is also reported as approaching 100% [26,27,30,32]. In Quebec, following introduction of SUAC as a first-tier screening test in 1998, 67/67 infants with TT1 were identified by their screening protocol [25]. In 2013, a group of European and Canadian expert clinicians recommended that SUAC be the primary marker for TT1 screening [17]. They were echoed in a North American consensus statement in 2017 [16].

There has been inconsistent implementation of effective NBS for TT1 in the United States, despite the condition being included in the Recommended Uniform Screening Panel (RUSP) since its inception in 2006 [18]. Major challenges to implementing SUAC testing into routine NBS in the US include lack of funding, need for additional laboratory staff and instrumentation, limited physical laboratory space, and concerns about assay validity [28]. Outside of Quebec, the earliest SUAC testing implementation reported was 2007 [28]. By 2014, 38 states included testing of SUAC as a marker of TT1 in their NBS protocols [28]. As of March 2020, there remain 3 states that have not incorporated SUAC quantitation in their NBS approach: Maryland, Oklahoma, and West Virginia.

Here, we present the case of an infant presenting with hyperinsulinemic hypoglycemia and subsequently critically ill with multiorgan failure, found to have TT1 caused by a novel pathogenic variant in FAH (c.1014 delC in exon 12; p.Cys 338 Ter) that was missed by NBS utilizing tyrosine quantification as the first-line disease marker. We detail the ensuing diagnostic odyssey and morbidity resulting from post-symptomatic diagnosis.

## 2. Case Report

The patient’s parent provided consent for publication of the following clinical data. The patient was a female of Ecuadorian heritage. She was spontaneously conceived and subsequently delivered at a community hospital in New Jersey at 36 2/7 weeks gestation to a 31-year-old gravida 2 para 2 via cesarean section. The pregnancy was complicated by a failed oral glucose tolerance test, though it was unclear from records if gestational diabetes management was initiated. Her Apgar scores were 9 and 9 at 1 and 5 min of life, respectively. Her weight and length were both in the 75th percentile for gestational age. Shortly after birth on her first day of life (DOL), hypoglycemia was noted with blood glucose measuring 12 mg/dL. Her hypoglycemia persisted and was managed with oral, then intravenous dextrose. Endocrinology was consulted and diagnostic laboratory testing demonstrated hypoketotic hypoglycemia with an inappropriately high insulin level given hypoglycemia (6.4 µIU/L in the setting of blood glucose < 50 mg/dL), low β-hydroxybutyrate (1.4 mmol/L; reference < 2.0 mmol/L) and a positive response to glucagon stimulation test, consistent with a diagnosis of hyperinsulinism. On DOL 14, diazoxide therapy of 10 mg/kg/day was initiated. On DOL 20, care was transferred to our center in Pennsylvania for additional evaluation and management of her hyperinsulinism. At that time, her diazoxide dose was 15 mg/kg/day and she required a continuous glucose infusion rate of 5 mg/kg/min.

With regards to NBS, the infant had a routine dried blood spot sent to the New Jersey state NBS laboratory on DOL 2 (Table 1), with normal results reported 2 days following sample receipt. This sample was analyzed for all conditions on that state’s NBS panel, including TT1. At the time, the screening protocol for TT1 was comprised of tyrosine MS/MS measurement. The patient’s tyrosine levels on the first NBS were within acceptable range at 151 µmol/L (reference cut-off < 280 µmol/L). On DOL 6, 13, and 18, repeat dried blood spot samples were sent due to the patient’s prematurity and only analyzed for congenital hypothyroidism and congenital adrenal hyperplasia (Table 1). Results from all screening were available to the patient’s care team at the time of her transfer to Pennsylvania. Following the patient’s TT1 diagnosis, her retained dried blood spot samples were retrospectively reanalyzed via MS/MS for both SUAC and tyrosine, with results shown in Table 1. SUAC from the first sample, sent on DOL 2 and subsequently stored under temperature-controlled conditions, was elevated at 5.23 µmol/L. New Jersey did not employ SUAC quantitation for TT1 screening, therefore there was no validated reference threshold at the time of this analysis. On adoption of SUAC-based screening in January 2020, the threshold was < 0.5 µmol/L. Finally, the New Jersey NBS laboratory was not blinded to the patient diagnosis at the time of reanalysis.

On DOL 23, the patient developed progressive abdominal distention and irritability with feeds. Abdominal imaging revealed a large stool burden, which partially improved following a glycerin suppository. Her clinical examination was otherwise unremarkable, and discharge was planned for the following day. However, on DOL 24, routine electrolyte assessment revealed severe hyponatremia of 112 mmol/L (reference: 135–145 mmol/L). Her mother commented that the patient had been sleepier than usual, prompting physical examination. She was somnolent but arousable to tactile stimulation, with mild facial edema, respiratory distress evidenced by intermittent intercostal retractions, and continued abdominal distention. Intravenous normal saline was initiated for gradual sodium replacement. When she subsequently developed hypotension requiring initiation of more aggressive fluid resuscitation, she was transferred to the Neonatal Intensive Care Unit for further management.

Following fluid resuscitation, hepatomegaly was noted. An abdominal ultrasound was obtained and was concerning for hepatic and renal echogenicity abnormalities. However, her critical illness prevented better characterization of these abnormalities with additional imaging. Her α-1-fetoprotein level was elevated at 164,000 ng/mL (reference: 0.6–77.0 ng/mL). Although her hypotension stabilized with multiple vasopressors, the infant’s course was further complicated by the development of fluid overload, pulmonary hypertension, acute kidney injury, and disseminated intravascular coagulation with bleeding from multiple sites. A head ultrasound revealed bilateral subdural hematomas. Her respiratory failure required intubation, and worsening ascites necessitated peritoneal drain placement.

The patient’s acute decompensation and liver failure lead her intensive care team to consider an inborn error of metabolism and the Metabolism team was consulted on DOL 26. Oliguria with significant gross hematuria complicated collection of urine for organic acids and SUAC quantitation. Meanwhile, hypotension, need for blood replacement products, and need for other laboratory evaluation important in stabilizing the patient delayed collection of the blood sample for plasma amino acids. Plasma amino acid testing run in-house was obtained on DOL 30 and reported on DOL 31. It was concerning for elevated plasma tyrosine (586.4 µmol/L; reference 22–102 µmol/L; 10.6 mg%) and phenylalanine (369.6 µmol/L; reference 23–95 µmol/L; 6.1 mg%). However, interpretation of plasma amino acid values was complicated by a generalized amino academia, consistent with known liver and renal dysfunction.

**Figure 1:**
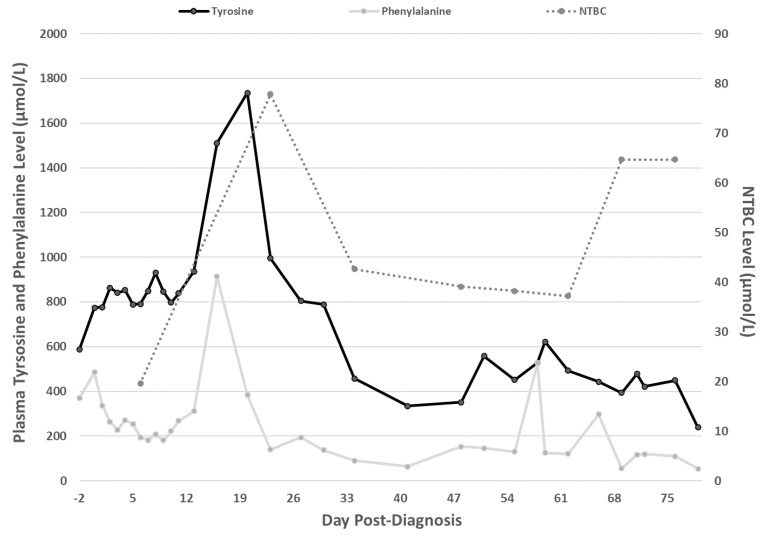
Patient tyrosine, phenylalanine, and 4-hydroxyphenylpyruvate dioxygenase inhibitor 2-(2-nitro-4-trifluoromethylbenzoyl)-1, 3-cyclohexanedione (NTBC) plasma levels over time. Normal tyrosine ranges between 22–102 µmol/L and normal phenylalanine ranges between 23–95 µmol/L. NTBC therapy was initiated on the first day following diagnosis using weight-based dosing. NTBC levels were quantified at Seattle Children’s Hospital Laboratory and are shown on the right y-axis. The NTBC therapeutic window is 40–60 µmol/L.

Diagnosis of TT1 was made when semi-quantitative urine organic acid analysis revealed high levels of tyrosine metabolites 4-hydroxyphenylacetate (2037 mg/g creatinine) and 4-hydroxyphenylpyruvate (161 mg/g creatinine) as well as the presence of SUAC (48 mg/g creatinine; reference undetectable). Protein was removed from her parenteral nutrition, and instead she received intravenous dextrose-containing fluids and lipids. NTBC pharmacotherapy (1 mg/kg/day) was initiated the following day (DOL 32). Soon after, rapid whole exome sequencing, obtained in the setting of multi-organ failure of unknown etiology, confirmed the diagnosis with a novel homozygous pathogenic nonsense mutation in *FAH* (c.1014 delC, p.Cys 338 Ter). With initiation of NTBC, the infant’s SUAC level normalized to undetectable on urine organic acid measurement within 3 days. Plasma SUAC measured via blood spot was within normal limits (1.2 nmol/mL; reference < 3 nmol/mL) 3 weeks following NTBC initiation and not quantifiable 2 weeks after that. As selective elimination of tyrosine and phenylalanine from parenteral nutrition was not possible, complete amino acids were gradually reintroduced to her nutrition on DOL 33. Adjustments were made to her nutritional management based on the sum of tyrosine and phenylalanine content as tyrosine levels rose, expected in the setting of NTBC (Figure 1**)**. Variations in plasma NTBC without changes in weight-based dosing were the result of challenges in weight-based dosing for a patient with profound ascites and fluid shifts who is also presumably undergoing some degree of growth. Routine ophthalmologic exams, initiated given risk for tyrosine corneal deposits, have remained normal to date.

**Figure 2:**
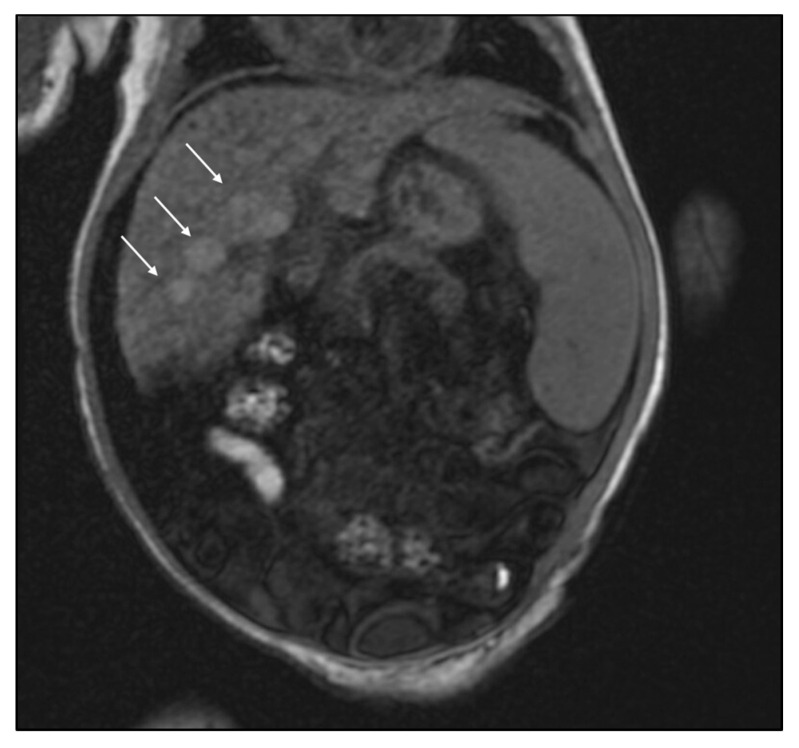
Non-contrast, T1-weighted abdominal MRI from DOL 68 showing the patient’s liver enlargement and irregular surface consistent with cirrhosis and multiple regenerative nodules (white arrows). The spleen is also enlarged, and there is abdominal distention secondary to ascites.

Gastroenterology was consulted to consider the utility of hepatic transplantation in this critically ill infant. Magnetic resonance imaging of the patient’s liver obtained once the patient had stabilized on DOL 68 revealed hepatomegaly and a nodular liver consistent with cirrhosis with multiple regenerative nodules but no masses suspicious for HCC (Figure 2). With stabilization, α-1-fetoprotein levels declined to 76,759 ng/mL 13 weeks post-diagnosis. As her respiratory status and fluid balance improved, the patient was extubated to non-invasive positive pressure ventilation on DOL 45. Despite adequate metabolic control of her TT1 with low phenylalanine and tyrosine levels, and NTBC levels within therapeutic range (Figure 1), her liver synthetic function failed to recover. The patient continued to struggle with ascites requiring continued peritoneal drainage, coagulopathy requiring frequent cryoprecipitate transfusions, thrombocytopenia, hyperbilirubinemia, and mild hyperammonemia. As a result, evaluation for hepatic transplant was initiated and the infant received an orthotopic LT from a deceased donor at 5 months of age. Her post-operative course was complicated by respiratory failure, temperature instability concerning for sepsis, and pancytopenia. She remained hospitalized in a critical care setting at the time of this report’s publication at 6 months of age due to ventilation needs. At that time, laboratory studies demonstrated normal liver parenchymal function, including coagulation studies, albumin, bilirubin, aspartate transaminase, alanine transaminase, and γ-glutamyl transferase. She has never been discharged from the hospital.

## 3. Discussion

This case underscores the importance of adherence to the NBS consensus statements for inclusion of SUAC as a primary marker for hereditary TT1 [16,17]. Screening for TT1 using tyrosine levels alone is known to result in false-negative cases, with at least two others in the literature [29,30]. Interestingly, reanalysis of this patient’s repeated newborn dried blood spot samples (Table 1) demonstrated tyrosine levels that surpassed the screening threshold by DOL 6 yet did not continue to rise. However, those dried blood spots, sent after the initial comprehensive NBS sample, were only analyzed for endocrine abnormalities and not amino acids. Although SUAC was consistently detectable on retrospective reanalysis (Table 1), its quantitation was not part of the New Jersey NBS protocol at that time.

Capturing TT1 patients during their pre-symptomatic phase and instituting early pharmacologic and dietary interventions is the goal of NBS [16,17,18]. There is evidence that this improves survival [13] and cognitive outcomes [21] while reducing the need for LT [11,13,19]. Although the retrospective nature of this case prevents certainty in what this infant’s outcome might have been in the case of a NBS-facilitated TT1 diagnosis, it remains likely that morbidity could have been prevented in the avoidance of an acute decompensation. The Quebec NTBC Study showed that during 5731 months of NTBC treatment, there were no hospitalizations for acute TT1 complications [13], suggesting that earlier initiation of NTBC could have prevented this infant’s multiorgan failure. Her false-negative NBS also delayed diagnosis; once TT1 was suspected, the patient’s critical illness delayed obtaining appropriate diagnostic samples.

Since the identification of this patient, beginning in mid-January 2020, New Jersey has implemented a SUAC-based TT1 screening program. Three states (Maryland, Oklahoma, and West Virginia) and many countries around the world do not utilize SUAC as a primary screening marker for TT1. With an incidence of the disease in the general population of about 1:100,000 [3], such policies likely result in rare but missed opportunities to attenuate morbidity and mortality for patients and their families. Furthermore, advances in molecular and genetic technologies over the past two decades may present new and exciting potential therapies for TT1, including enzyme replacement therapy, gene therapy, and genome editing [34]. These options, while not yet a reality, may mean that babies born and diagnosed through NBS today may have therapeutic modalities available to them that continue to improve outcomes, making early, accurate, pre-symptomatic recognition of the disease more important.

Here, we report an infant with molecularly and biochemically verified TT1 whose NBS was reported by the reported by the state’s NBS laboratory as “normal.” “False negative” results are results in which the condition being tested is present, but not detected. False negatives can occur for analytic reasons (e.g., the analyte is present, but not detected), but can also occur when the analyte under study is not sensitive for the condition. In this case, the New Jersey NBS correctly reported a normal tyrosine level, and one may argue that to practitioners who are knowledgeable about the negative predictive value of tyrosine as a sole analyte for the diagnosis of TT1, this is the expected result. While this is true, such information regarding such limitations is not disseminated to healthcare providers outside of the metabolism specialty. This is problematic: a screen reported as normal will have results sent to an infant’s primary care provider/team, who may not fully understand the methodology of the test. If no distinction is made on the report between use of tyrosine and SUAC for TT1 screening, practitioners in catchment areas that include multiple states employing multiple NBS modalities may be tempted to consider universal screening for TT1 across the United States as equivalent. Labeling the case as a false negative reminds the practitioner of the need for vigilance, in verbiage familiar to clinical practice.

It is unrealistic to expect non-metabolic physicians to be aware of the sometimes-nuanced differences between NBS methodologies and diagnostic yields that can exist both between municipalities and between different screening laboratories. For these clinicians, the case underscores the importance of early consultation with a metabolic specialist rather than reliance on a negative NBS result. For metabolic specialists, particularly those in multi-state catchment areas, it underscores the importance of familiarity with methodologic screening differences.

Although SUAC quantitation is an important part of screening for TT1 with high sensitivity [33], it is imperative to note that no screening tool can be perfect. Indeed, Blackburn et al. reported a family with a homozygous variant in *FAH* affecting the catalytic pocket of the enzyme without affecting tyrosine or SUAC levels [35]. Three affected individuals presented in infancy with hepatosplenomegaly and cirrhosis, progressing to HCC in childhood. The family was ultimately diagnosed via whole exome sequencing, after urine organic acid levels were normal and SUAC levels were undetectable. One sibling had tyrosine levels within normal range, and another had levels only mildly elevated (212 nmol/mg Cr, where the upper limit of normal was 208 nmol/mg Cr). Thus, while a SUAC-based screening program likely would have avoided morbidity for our patient given the high SUAC levels present on reanalysis of her filter paper (Table 1), it cannot do so for all patients. As a result, maintaining familiarity with the TT1 phenotype and suspicion for inborn errors of metabolism in infants with liver pathology, remain important tools in any clinician’s toolbox.

Finally, hyperinsulinism can be an early symptom of TT1. Children with TT1 presenting in the neonatal period commonly have hypoglycemia [8] and pancreatic islet cell hyperplasia has been identified in a number of patients [36,37]. In one retrospective review of 25 infants, three met clinical criteria for hyperinsulinism with hypoglycemia and inappropriately elevated insulin or C-peptide [38]. The patients either presented in liver failure (2/3) or were diagnosed pre-symptomatically via NBS [38]. They were successfully treated with diazoxide and chlorothiazide [38]. With resolution of their hypoglycemia, they were able to be weaned off of treatment, suggesting a transient nature to their hyperinsulinism [38]. However, the described patients carried a known TT1 diagnosis and were also treated with NTBC [38], making the time course of this feature as a function of disease versus a function of treatment unclear. To our knowledge, this is the first patient whose initial presenting symptom of TT1 was hyperinsulinemic hypoglycemia, suggesting it occurs as a product of the disease rather than NTBC treatment. It is thus important to recognize that hyperinsulinism can be a feature of TT1, particularly in infants presenting with liver dysfunction and hypoglycemia.

## 4. Conclusions

Here, we report the case of an infant presenting with hyperinsulinism and developing multi-organ failure who harbored a novel homozygous pathogenic nonsense mutation in *FAH* (c.1014 delC; p.Cys 338 Ter) consistent with TT1. This was not identified via NBS, which relied on tyrosine rather than SUAC quantitation. Her resulting morbidity underscores that SUAC should be the universal first-line marker for NBS for TT1. The case also illustrates that NBS is important, but not infallible. Medical teams must keep a broad differential diagnosis even in the setting of a seemingly reassuring NBS. We also remind clinicians that neonates with TT1 can present with hypoglycemia and meet clinical criteria for hyperinsulinism.

## Figures and Tables

**Table 1:** Tyrosine quantitation from the patient’s original DOL 1 dried blood spot analysis (top line, bolded) with retrospective re-analysis of stored blood spot samples quantitating both tyrosine (cutoff <280 µmol/L) on the subsequent 3 samples and SUAC on all 4 samples. There was no validated cutoff for SUAC given that it was not part of New Jersey’s TT1 screening protocol at the time. Reported screening results from the New Jersey NBS Laboratory and comments on results are shown on the right. The patient’s repeated screening test on DOL 18 was flagged for low T4 (8.0 µg/dL with cutoff >14.5 µg/dL).

Age at Collection	Tyrosine (µmol/L)	Succinylacetone (µmol/L)	Reported Result	Comments
1 day 9 h	151	5.23	Normal	Full screen
6 days 2 h	437	4.65	Normal	Only screened for congenital hypothyroidism and congenital adrenal hyperplasia
13 days 2 h	312	5.57	Normal	Only screened for congenital hypothyroidism and congenital adrenal hyperplasia
18 days 20 h	409	10.34	Abnormal	Low T4, TSH within acceptable limits
